# High Area Capacity Lithium-Sulfur Full-cell Battery with Prelitiathed Silicon Nanowire-Carbon Anodes for Long Cycling Stability

**DOI:** 10.1038/srep27982

**Published:** 2016-06-20

**Authors:** Andreas Krause, Susanne Dörfler, Markus Piwko, Florian M. Wisser, Tony Jaumann, Eike Ahrens, Lars Giebeler, Holger Althues, Stefan Schädlich, Julia Grothe, Andrea Jeffery, Matthias Grube, Jan Brückner, Jan Martin, Jürgen Eckert, Stefan Kaskel, Thomas Mikolajick, Walter M. Weber

**Affiliations:** 1 Namlab gGmbH, 01187 Dresden, Germany; 2 Center for Advancing Electronics Dresden (CfAED), TU Dresden, Dresden, Germany; 3 Fraunhofer Institute for Material and Beam Technology (IWS), Winterbergstr. 28, 01277 Dresden, Germany; 4 Department for Inorganic Chemistry I, TU Dresden, Dresden, Germany; 5 Leibniz Institute for Solid State and Materials Research (IFW) Dresden e.V., Helmholtzstr. 20, 01069 Dresden, Germany; 6 Institut für Werkstoffwissenschaft, TU Dresden, Helmholtzstr. 7, 01069 Dresden, Germany; 7 Erich Schmid Institute of Materials Science, Austrian Academy of Sciences, A-8700 Leoben, Austria; 8 Department Materials Physics, Montanuniversität Leoben, Jahnstraße 12, A-8700 Leoben, Austria; 9 Institute of Semiconductor and Microsystems Technology, TU Dresden, 01062 Dresden, Germany

## Abstract

We show full Li/S cells with the use of balanced and high capacity electrodes to address high power electro-mobile applications. The anode is made of an assembly comprising of silicon nanowires as active material densely and conformally grown on a 3D carbon mesh as a light-weight current collector, offering extremely high areal capacity for reversible Li storage of up to 9 mAh/cm^2^. The dense growth is guaranteed by a versatile Au precursor developed for homogenous Au layer deposition on 3D substrates. In contrast to metallic Li, the presented system exhibits superior characteristics as an anode in Li/S batteries such as safe operation, long cycle life and easy handling. These anodes are combined with high area density S/C composite cathodes into a Li/S full-cell with an ether- and lithium triflate-based electrolyte for high ionic conductivity. The result is a highly cyclable full-cell with an areal capacity of 2.3 mAh/cm^2^, a cyclability surpassing 450 cycles and capacity retention of 80% after 150 cycles (capacity loss <0.4% per cycle). A detailed physical and electrochemical investigation of the SiNW Li/S full-cell including in-operando synchrotron X-ray diffraction measurements reveals that the lower degradation is due to a lower self-reduction of polysulfides after continuous charging/discharging.

Lithium-sulfur batteries are pointed out as one of the most promising systems for advanced application in automotive or stationary electrical energy storage. The electrode components are predicted as lower in price and naturally highly abundant and therewith uncritical for shortage of resources. Introduction into the market has been prevented by limited stability and capacity as well as the absence of a suitable electrolyte when using full-cells with carbon-sulfur cathode, as carbonate-based electrolytes cannot be used due to their reaction with the polysulfides. Further on, present Li/S batteries employ a metallic Li foil as anode material resulting in undesired formation of cell safety critical Li dendrites. The use of a metallic Li anode can result in the loss of active material at the positive electrode, enhanced reactions with the electrolyte or short-circuiting of anode and cathode through the separator^1^. These points are addressed as key factors for high battery performance, long lifetime and safety. Silicon is one of the most promising anode materials replacing metallic Li due to a maximum specific capacity of 3579 mAh/g at room temperature^2^ but is accompanied by a large volume change upon lithiation and de-lithiation. To overcome stress related fracturing, several Si-based nanostructures^3^ that by their intrinsic geometry and neighboring free expansion volume allow an enhanced mechanical stress relaxation, like nanoparticles^4^,^5^,^6^, nanowires^7^,^8^,^9^,^10^,^11^,^12^,^13^, microwires^14^ and nanotubes^15^,^16^,^17^,^18^, have been studied in battery setups. Further on, prelithiated Si nanostructures allow the insertion of Li independent of the chosen cathode material^6^,^8^,^19^,^20^.

As an ideal nanostructure to ensure both the possibility of free expansion and relaxation of Si as well as a good electrical contact to a carbon current collector, pure Si nanowires (SiNWs) have been investigated in half-cell setups as a promising solution to address both issues^12^,^21^. Recently, the SiNWs are grown by using planar sputtered Au layers to create Au nanoparticles (NPs) on a 3D surface^11^. A 3D SiNW growth with commercially available Au NPs has been reported for a 2^nd^ generation Li ion battery setup with LiFePO_4_ cathode and LiPF_6_ electrolyte with a maximum capacity of 100 mAh/g_LiFePO4_^21^. Nevertheless, Si appeals to be used as anode in high capacity lithium-sulfur systems since its high theoretical specific capacity is related to an insertion of 15 mole Li to 4 mole Si (Li_15_Si_4_). This high Li content compared to carbon with 1 mole Li per 6 mole C helps to build thin electrodes and to allow an easier balancing of the battery cell with excess Li of more than 2000% related to the sulfur mass^22^.

In this publication, we present a balanced Li/S battery with high capacity and long cycling stability using a prelithiated SiNW/C composite as anode material (6.0 mAh/cm^2^) and a S/C composite for the positive electrode (2.4 mAh/cm^2^) to overcome the battery failure due to typical Li dendrite formation resulting from state-of-the-art metallic Li foils. The Li excess in the prepared full-cell is limited to 200% of the cathode capacity, which allows an enhanced cycling stability^22^. Our full-cell shows with 460 cycles a 30 times higher cycling stability compared to previously reported Li/S batteries with SiNWs^7^ and has a high specific capacity of 1.6 mAh/cm^2^ (714 mAh/g_sulfur_) after 200 cycles. The conformal and heterogeneous integration of SiNWs with sufficient Si loading (2.2 mg/cm^2^) on the light-weight and conductive carbon mesh is the key element to achieve a high cycling stability and capacity. To enable the nanowire growth on the three-dimensional carbon network, a novel versatile gold ‘Pechini’ precursor was developed giving very homogenous and dense seed particle coverage. Physical analysis including in-operando synchrotron x-ray diffraction (SXRD) elucidates the mechanisms for the cell stability. The integration of SiNWs on carbon meshes is a highly promising approach to achieve high capacity batteries without elemental Li in combination with sulfur cathodes and ether-based liquid electrolytes to gain significantly improved cycling stability compared to known samples from literature^7^,^23^.

For the previously addressed tasks, the manuscript is divided into three parts. In the first part, morphology and crystallinity of the SiNW/C composite was analyzed before integration in a battery setup. The second part discusses the electrochemical investigations of the SiNW/C composite in half-cell assemblies vs. Li/Li^+^ with subsequent in-operando as well as post-mortem characterization of morphology and crystallinity to elaborate the basic capacity fading mechanisms. The third part covers the integration of SiNW/C anodes in high capacity balanced Li/S full-cells by means of in-operando synchrotron X-Ray diffraction (SXRD) measurements and detailed electrochemical characterization.

## Results and Discussion

### Structural characterization of the as-grown SiNWs

**Figure 1:**
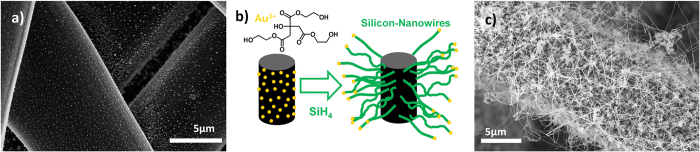
(**a**) Carbon fibers with three-dimensionally deposited Au nanoparticles. (**b**) Schematic view of the anode assembly based on the growth of Si nanowires on top of a single carbon fiber. The Au nanoparticles are the catalysts for the VLS growth of diluted SiH_4_ at 420 °C. (**c**) Carbon mesh completely covered with Si nanowires with a Si loading of more than 3.0 mg/cm^2^ prior to integration in Li half-cell.

The preparation of the SiNW/C composite electrodes is depicted in Fig. 1. The SiNWs are grown selectively with the prepared Au nanoparticles as catalyst seed (Fig. 1a) via the vapor-liquid-solid (VLS) mechanism and SiH_4_ as the Si precursor gas on the carbon mesh. A detailed description of the novel Au nanoparticle fabrication can be found in the Suppl. Info. (Figs S1–3). Figure 1b schematically describes the VLS growth of SiNWs on the carbon fibers. Figure 1c presents a representative sample with very high SiNW loading of more than 3 mg/cm^2^ on a single carbon fiber. The amount of Si is reproducibly tuned by deposition time as well as SiH_4_ dilution in H_2_ to optimize the Si content of the negative electrode composite shown in Krause *et al*.^24^. Comparable SiNW loadings on carbon meshes have been recently reported by Peled *et al*.^21^ using commercially available Au nanoparticles. Nevertheless the development of a stable and recyclable Au precursor enables a resource efficient gold catalyst seed application on scalable substrates in this work. For the assembly of a well-balanced full-cell, a SiNW/C composite as negative electrode can be prepared matching the high S/C positive electrode composite.

**Figure 2:**
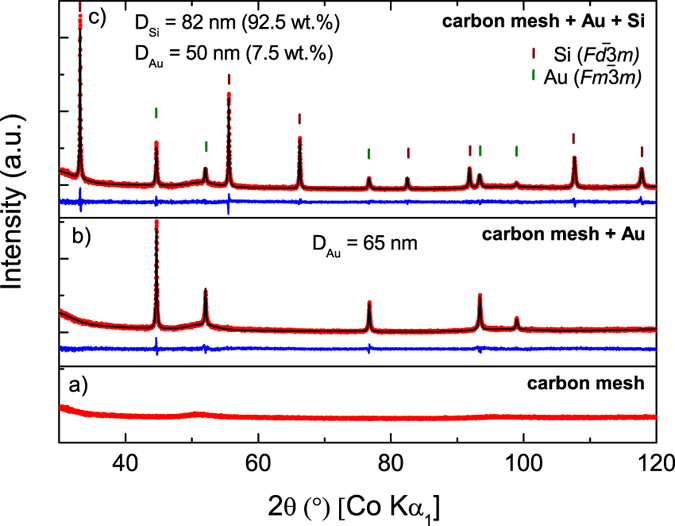
XRD pattern (red) of the carbon mesh (**a**), the Au-treated carbon mesh (**b**) and the final SiNWs (**c**). Crystallite sizes and phase contents of the crystalline phases are listed in the graphic determined by a Rietveld analysis. The result of the Rietveld analysis is depicted as the calculated pattern in black. The difference between calculated and measured pattern is plotted in blue.

The convenient sample structure enables an excellent insight with x-ray diffraction analysis. Figure 2 summarizes the structural properties of each step of the SiNW/carbon composite electrode preparation prior to lithiation with X-ray powder diffraction measurements. The bare substrate shows the signature of an amorphous carbon layer with a broad signal with a maximum at 2θ = 51° (Fig. 2a). After the deposition of the Au layer on the carbon fibers and the formation of Au nanoparticles on the surface, reflections appear in the pattern (Fig. 2b) corresponding to the cubic structure of Au with space group 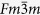. After the growth of the SiNWs, distinct reflections of crystalline Si with the space group 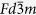 appear while Au becomes the minor phase as expected (Fig. 2c). An averaged Au crystallite size of 65 nm is determined for Au, which is decreased to approximately 50 nm after SiNW growth. The observed crystallite size for the crystalline Si is calculated to 82 nm in average. High-resolution TEM bright field images of the samples with as-grown crystalline SiNWs are shown in the Suppl. Info. (Fig. S4) including EDXS analysis (Fig. S5).

### Electrochemical characterization of SiNW electrodes versus lithium

**Figure 3:**
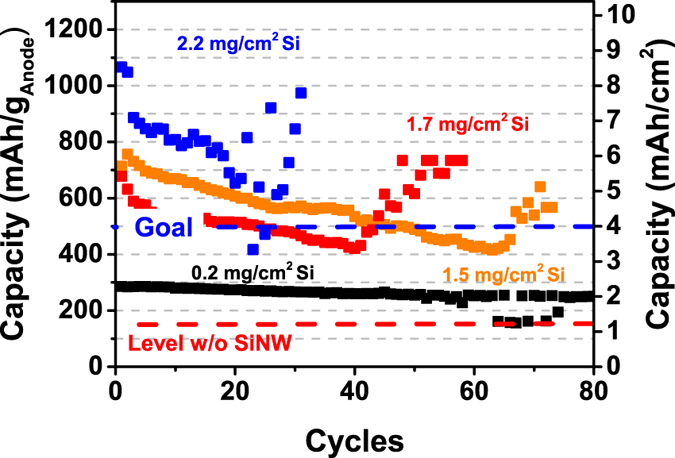
Cycling Stability of anodes of SiNW in carbon mesh versus lithium for different Si loadings. The 1^st^ cycle of a cell with 2.2 mg/cm^2^ SiNW loading shows an excellent capacity of up to 9 mAh/cm^2^.

Prior to integration in Li/S full-cells, half-cells with SiNW/C composite electrodes vs. elemental Li are studied in the ether- and lithium triflate-based electrolyte to understand the electrochemical behavior of SiNW electrodes. The electrochemical test of the composite as positive electrode reveals outstanding high areal capacities up to 9 mAh/cm^2^ (Fig. 3). When compared to the areal capacity of a pure carbon mesh (1.2 mAh/cm^2^), SiNWs drastically increase the areal capacity due to the high theoretical gravimetric capacity of Si (3579 mAh/g_Si_ for Li_15_Si_4_). For practical applications of Li/S cells, an areal capacity of 6.0 mAh/cm^2^ is required to reach energy densities of commercial lithium-ion batteries^25^. This outstanding high value is reproducibly exceeded by the SiNW composite anodes presented in this work. Note that the presence of Au particles makes a negligible contribution to the charge capacity, e.g. shown by Chan *et al*.^26^. To further reduce costs, SiNWs can also be grown with many different catalyst metals, such as Cu, Ti, Al, In etc. shown by *Schmidt et al*.^27^.

Figure 3 also depicts that an increase in SiNW mass loadings leads to lower cycle stability of the Li half-cells. The high capacities of the cells result in high charge/discharge currents when using a constant rate of C/5. It is known, that high currents promote Li dendrite formation of metallic Li and induce active material loss during cycling^28^,^29^,^30^. Other reasons of decreasing capacity or cell failure may be caused by electrolyte depletion due to an unstable SEI^31^,^32^,^33^ or due to the loss of electrical contact between the SiNWs and the carbon current collector during cycling. However, in the lithium-sulfur full-cells described later, several mechanisms can be disregarded due to the absence of elemental Li.

**Figure 4:**
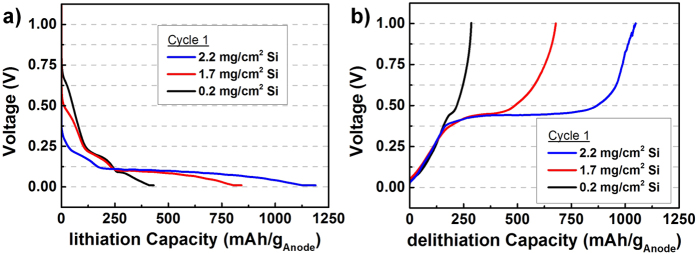
Voltage Profile in the 1st cycle using a carbon mesh with different mass loading of SiNWs in the half-cell setup during lithiation (**a**) and delithiation (**b**) of Si.

The influence of different Si mass loadings on the capacity is derived from the voltage profiles of the half-cells in Fig. 4. Almost only the carbon influences the reachable capacity of up to 250 mAh/g, when a carbon mesh with a low Si loading of 0.2 mg/cm^2^ is used. Higher Si loadings show a long flat voltage plateau at approximately 0.11 V during lithiation (Fig. 4a). The appearance of this plateau represents the formation of lithiated amorphous Si, which is the lithiation potential of pure crystalline Si from SiNWs^34^,^35^. The following gradual dropping to 0.01 V may be related to the lithiation to Li_15_Si_4_ as the lithium-richest compound to be electrochemically formed at room temperature^2^,^36^. The delithiation of the Li-Si alloy is visible due to a plateau in Fig. 4b at about 0.45 V. The appearance of this plateau stands in particular for delithiation of Li_15_Si_4_ and the coexistence with an amorphous Li_z_Si phase^2^,^35^,^36^.

### Structural characterization of cycled SiNWs electrodes

**Figure 5:**
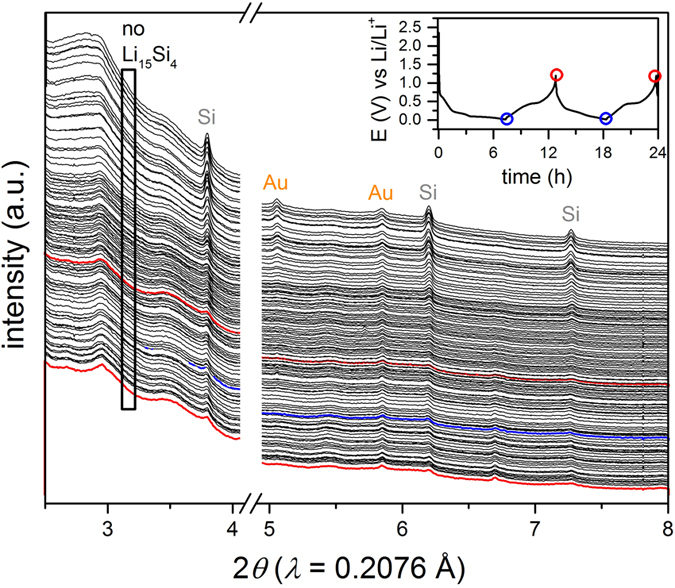
In-operando synchrotron powder diffraction data of the SiNW/C composite electrode cycled vs Li/Li^+^ (half-cell) at 0.5 mA/cm^2^. Corresponding electrochemistry with assigned position of the colored XRD pattern (inset top right). Si and Au pattern diminish during first charging due to amorphization with Li insertion. Other peaks can be attributed to e.g. LiOH formation or inactive battery components.

To study the degradation phenomena of the SiNW/C composite electrode, in-operando as well as post-operation analysis was carried out in the half-cell setup. In-operando SXRD was used to record the first cycles and observe the structural changes of the SiNWs as shown in Fig. 5. The Si reflections successively diminish distinctly depending on time related to an amorphization process at simultaneously increasing the Li content during charging. Some residues of crystalline Si and Au remain at the end of discharge as a result of incomplete lithiation owing to the relatively high current rate of 0.5 mA/cm^2^ or insufficient electrical contact. These high current rates have been first reported for in-operando Si lithiation here, but are essential to understand degradation mechanisms for product-oriented application. No crystalline Li_15_Si_4_ is observed at the end of the lithiation process^37^, typically causing a degradation of the Si anodes^36^,^38^,^39^. This result may explain the long-term cyclability of our samples^40^. As the SiNW showed continuous capacity loss at this current rate (Fig. 3), crystalline Li_15_Si_4_ is excluded as the major reason for degradation in our experiments.

**Figure 6:**
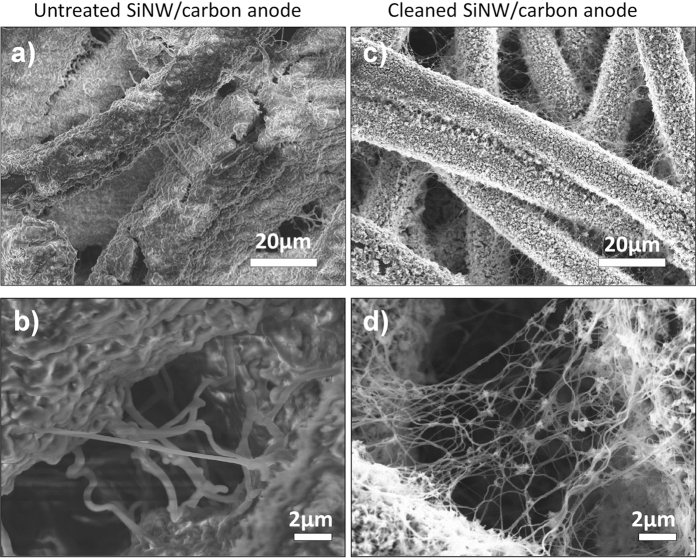
SEM investigation of cycled SiNW anodes. (**a**,**b**) A thick insulating SEI is formed on top of the carbon mesh and completely covers the SiNWs. (**c**,**d**) After removal of the SEI with diluted HNO_3_, free-standing nanowires are visible and are still attached to the carbon surface.

After ten charge and discharge cycles, a coin cell was dismantled and the SiNW/C electrode (Si loading approx. 1.5 mg_Si_/cm^2^) was removed for analysis. The sample was rinsed in diluted HNO_3_ to remove electrolyte residues. Figure 6 shows the SEM images of this sample: Most of the nanowires are still attached to the carbon fibers. The images illustrate an intact electrical contact of the SiNWs with the substrate resulting in long cycling duration. This finding is in contrast to results presented by Chan *et al*.^26^ dealing with the hypothesis of cell failures due to structural disintegration of the SiNWs. The cycled sample shows undulated Si nanowires, which are attributed to the final relaxed amorphous state originating from the initially single crystalline Si nanowires. This bending upon lithiation starting with the first cycle is in accordance with literature^41^,^42^.

**Figure 7:**
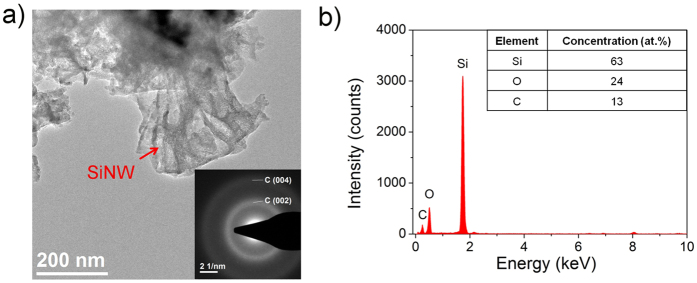
TEM analysis of the cycled SiNWs on the carbon support. (**a**) Bright-field images and SAED pattern and (**b**) the corresponding EDX measurement of the analyzed area.

Additional structural information of the cycled SiNW/C composite is shown in Fig. 7. Figure 7a depicts a bright-field TEM image of agglomerated SiNWs verified by the high concentration of Si in the EDX spectrum (Fig. 7b). The SAED patterns of the SiNW remains reveal no crystalline Si indicating amorphous Si. The complete amorphization of the crystalline SiNWs in the first lithiation cycles supports the in-operando measurements in Fig. 5. The SiNWs are considerably deformed as a result of the enormous volume change during cycling. High concentrations of oxygen are attributed to the exposure to air and the treatment with HNO_3_ to remove the interfering SEI after disassembling. The Debye-Scherrer rings in the SAED pattern in Fig. 7a represent both amorphous SiO_2_ as result of the etching process and amorphous carbon as it is characteristic for the supporting mesh.

### Characterization of prelithiated SiNWs in a full-cell setup

Previous half-cell tests versus Li/Li^+^ help to identify the basic working principles of the anode material and allows to compare the results to literature. Recently, SiNW/C composite negative electrodes with comparable capacity have been reported for LiFePO_4_ 2^nd^ Generation batteries^21^. In this work, prelithiated SiNW/C composite electrodes have been integrated in high capacity Li/S batteries to replace Li metal as only negative electrode with sufficient capacity. The antecedent prelithiation of SiNWs in a separate half-cell has the advantage to compensate the Li loss due to SEI formation in the first cycles^19^,^20^. Electrochemical testing of the composite electrodes in a Li/S full-cell exposes the real properties of the negative electrode material and provides the necessary data for introduction into application without the obstacle of Li dendrite formation. These results give valuable information primarily on the Li transport mechanism in Li/S cells.

**Figure 8:**
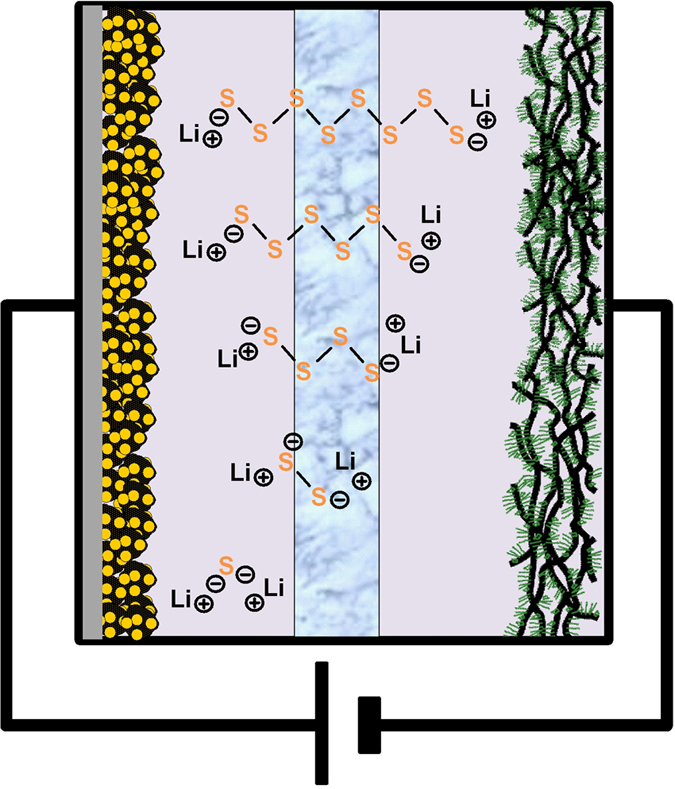
Scheme of Li/S full-cell with SiNW/C anode, S/C cathode and separator.

In contrast to Li-ion cells or to the Si-Li system, where only Li ions migrate between the electrodes, in Li/S cells the charge is carried by lithium-polysulfides. During discharging, sulfur is dissolved by the formation of long chained polysulfides. After reduction of the chain length to at least Li_2_S_4_, the final product of electrochemically-driven reaction is Li_2_S. During charging, lithium remigrates towards the negative electrode and sulfur is formed in the reverse reaction^43^,^44^,^45^. A scheme of the used Li/S full-cell assembly with S/C cathode and SiNW/C anode is presented in Fig. 8.

### In-operando synchrotron powder diffraction of prelithiated SiNW/S full-cell

**Figure 9:**
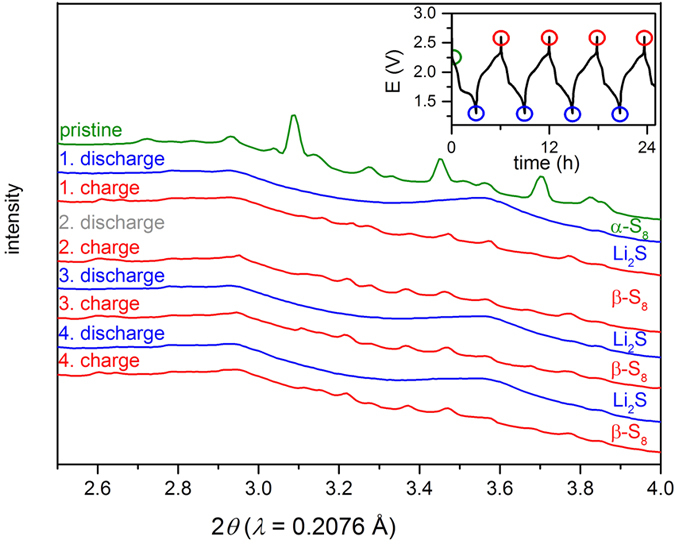
In-operando synchrotron powder diffraction data of the prelithiated SiNWs cycled vs S (full-cell) at 1/5 C. Corresponding electrochemistry with assigned position of the colored XRD pattern (inset top right). The diffraction pattern distinctly changes during the 1^st^ charge/discharge cycle from α-sulfur to *β*-sulfur.

In order to study the initial degradation phenomena, the SiNW Li/S full-cell was cycled for four times at a rate of 1/5 C and simultaneously diffraction patterns have been recorded every 10 min. Conventional Li/S half-cell batteries were investigated by in-operando diffraction previously^46^,^47^,^48^,^49^. Since the degradation of the Li metal electrode by migrating polysulfide species can considerably affect the degradation of Li/S batteries^22^, an investigation of a sulfur positive electrode vs. lithiated SiNWs by in-operando XRD measurements is of vast interest. The electrochemical results and the corresponding synchrotron diffraction pattern are demonstrated in Fig. 9. The entire waterfall plot of the measurements is found in the supplementary information (Fig. S6).

Initially, α-sulfur with space group *Fddd* is present as typically stable phase at ambient temperature in the positive electrode composite (Fig. 9a green pattern). No distinct crystalline Si reflection is observed in the initial pattern after the pre-lithiation process (Fig. 5). During the first discharging, the α-sulfur phase disappears as a result of the lithiation of sulfur and complete dissolution to polysulfides. At the end of the first discharge cycle Li_2_S appears with a broad {111} reflection. During recharging, the disappearing Li_2_S {111} reflection indicates a reversed reaction from Li_2_S to soluble polysulfides. Interestingly, at the end of the second charging, sulfur reconstitutes in its monoclinic β-allotrope with space group *P2*_*1*_*/c*. The structure model of β-sulfur was refined by the Rietveld method according to the diffraction data (see Supp. Info. in Fig. S7). At this stage, the entire process is highly reversible over at least four cycles with the final products Li_2_S and β-sulfur. No other crystalline phases are present in the investigated diffraction range. The results agreed with previously reported results for Li/S batteries^46^,^47^,^48^. The reflection intensities of Li_2_S and β-sulfur remain almost unchanged in each cycle which points to low self-discharge and low sulfur consumption on the Si negative electrode surface. This finding may be attributed to the lower reactivity of Si and its SEI compared to Li metal as shown for Si nanoparticles by Jaumann *et al*.^4^. Importantly, this data supports the fact that the lower self-reduction of polysulfides results in a lower degradation of our full-cell setup highlighting the relevance of this full-cell for real application.

### Cycling stability of SiNW/C anode vs S/C cathode

**Figure 10:**
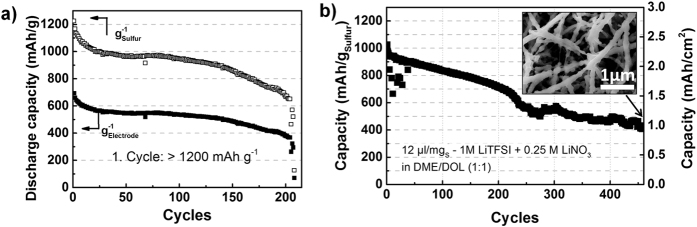
Comparison of the cycle performance of sulfur cathode versus (**a**) Li foil with cell failure after 200 cycles due to Li dendrite formation and (**b**) including SiNW anode, which show slow capacity decay but without complete cell failure up to 460 cycles. (Inset) SEM image of SiNW/C anode after 460 cycles.

To examine the long-term properties of lithiated SiNWs, a separate full-cell-test versus a sulfur positive electrode (2.4 mAh/cm^2^) was carried out. Chakrapani *et al*. showed that limiting the anode capacity promotes stable cycling performance^9^. Therefore, carbon meshes with a moderate Si loading of approximately 1.5 mg_Si_/cm^2^ (6.0 mAh/cm^2^) were chosen as anode materials for a balanced full-cell. Figure 10 compares the cycling performance of the sulfur positive electrode versus (a) Li and (b) the SiNW/C composite anode. Note that the capacity of the SiNW Li/S full-cell is limited by the S/C composite cathode due to the excess of Li from the prelithiated SiNWs. The capacity of 1027 mAh/g_sulfur_ corresponds to an energy density of 410 Wh/kg including the active components, the current collector and the separator. The excess of Si plays only a minor part in the energy density of the full-cell. Because of the high specific capacity of Si, a small extra amount of Si results in a remarkably higher negative electrode capacity, while the change to the weight is negligible compared to the other components.

Until cycle 150 the metallic lithium and the SiNW anodes show a similar capacity, with two exceptions. The first difference is faster degradation of cells with Li negative electrode during the first 25 cycles, possibly due to formation of Li dendrites or irreversible polysulfide dissolution. The second difference is the appearance of a capacity plateau with a metallic lithium anode until cycle 150, while SiNW anodes exhibit a gradual degradation with increasing number of cycles. This behavior is most likely caused by an irreversible electrolyte decomposition and continuous SEI formation at the SiNW surface. A progressive degradation of electrical contact of the SiNWs during lithium insertion has not been observed in the first cycles in Figs 5 and 6. As it is shown in Fig. 10b (Inset), the stability of the nanowire structures on carbon is assured beyond 460 cycles in the Li/S full-cell. Nevertheless, loss of electric contact as fading mechanism cannot be explicitly excluded in the long term measurements.

After 150 cycles the degradation of the cells with elemental Li foil as anode is accelerated until complete breakdown and cell failure occurs due to Li dendrite formation causing a short circuit between the electrodes. In the case of the full-cell with a SiNW-based anode, the short circuit is avoided even with fading capacity. After complete loss of SiNW capacity, the carbon mesh still can reversibly insert Li ions. Since the carbon mesh contributes with approx. 1.2 mAh/cm^2^ to the overall capacity (Fig. 3), the cell capacity is mainly shaped by the electrochemical property of carbon. For a description of the long cycling performance of carbon vs. a sulfur positive electrode, the reader is forwarded to Brückner *et al*.^22^.

**Figure 11:**
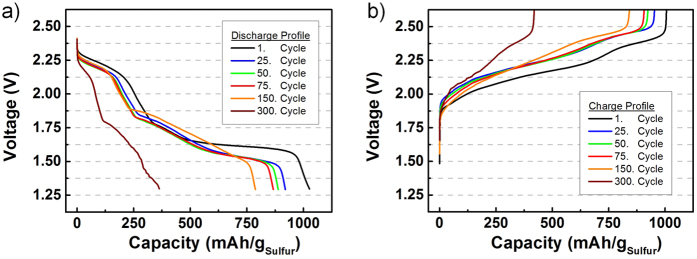
Voltage profiles for different cycles of lithium-sulfur-cell with SiNW anode: (**a**) discharge profiles and (**b**) charge profile.

The voltage profiles in Fig. 11 demonstrate the contribution of the carbon mesh during lithiation and delithiation. While the first discharge curve is characterized by a flat plateau at approximately 1.58 V, corresponding to the delithiation of Si, the plateau extension is reduced with every further cycle and the cell voltage decreases below 500 mAh/g_sulfur_. The charging profiles obviously remain unchanged during cycling, but an increased charge voltage may result from a loss of active material or the presence of an insulating layer on the electrode surfaces. Discharging as well as charging profiles at cycle 300 indicate that the carbon mesh is still working as an intercalation negative electrode for lithium ions and thereby allows a continuously and stable operating lithium-sulfur battery.

Even if the carbon mesh plays an important role in a Li/S full-cell using SiNW in carbon as an anode material, the advantages of Si are obvious:

- The anode capacity is significantly enhanced above pure graphite capacity for over 450 cycles.
- The use of potentially dangerous lithium is avoided.
- Cell failure due to lithium dendrite formation short-circuiting the electrodes is avoided.
- There is no need for a copper current collector, which reduces the inactive material mass of the cell.

The Li/S battery is limited by the S/C cathode not by the prelithiated SiNW/C composite anode as the anode provides an excess of Li for full charge/discharge cycles. The full-cell uses a standard setup with a commercial separator and LiTFSI based electrolyte. As the lithium polysulfide migration has been pointed out as major reason for SEI formation and electrolyte decomposition, further optimization in hindering the migrating polysulfide species would directly increase the cycling stability of this Li/S battery setup. Additionally the ‘Pechini’ precursor developed for versatile Au nanoparticle formation on various substrates allows a transfer of SiNW growth to other 3D substrates with optimized weight and porosity to further reduce the amount of electrolyte and an increased energy density of fully integrated Li/S battery cells for electro mobile applications.

In summary, this publication shows a new set of material and cell concepts for the Li/S battery system with high energy densities and long cycling stability with absence of elemental Li foil. Cathodes with high sulfur content made of carbon composites with a defined porosity (>65 wt.-% in carbon) and high specific capacity above 1200 mAh/g_sulfur_ (1000 mAh/g after 100 cycles) were prepared. The versatile gold precursor developed for a homogenous Au layer deposition allowed an adjustable Si loading to achieve balanced SiNW/C composite electrodes. The CVD SiNW growth occurred directly on the surface of the carbon fiber with a high area capacity of up to 9 mAh/cm^2^ (4 mAh/cm^2^ after 50 cycles). The result was a SiNW Li/S full-cell with an areal capacity of 2.4 mAh/cm^2^, a cyclability surpassing 450 cycles and capacity retention of 80% after 150 cycles (capacity loss lower than 0.4% per cycle). A low degradation due to a low self-reduction of polysulfides during charging/discharging is revealed with detailed in-operando synchrotron measurements, while other fading mechanisms could be excluded in the SiNW Li/S full-cell setup. The integration of SiNWs on carbon meshes is a promising approach for achieving high capacity batteries in conjunction with sulfur cathodes to avoid dendrite formation and gain improved cycling stability.

## Methods

### SiNW/carbon composite electrode preparation

Commercially available carbon fiber networks were used as a three-dimensional conducting substrate for SiNW deposition (Freudenberg FCCT SE & Co. KG, area weight 6.5 mg/cm^2^, size 3 × 4 cm^2^)^22^. A slightly modified noble metal ‘Pechini’ precursor based on citric acid, ethylene glycol and HAuCl_4_ solution was applied for conformal deposition of a thin layer of Au on the carbon mesh fibers (see Suppl. Info. for detailed description).

In order to remove all organic residues that could potentially contaminate the catalyst for successful nanowire growth, the substrates with Au nanoparticles where exposed to pure H_2_ prior to SiNW growth for 5 min at 420 °C in a chemical vapor deposition (CVD) vacuum furnace (ATV GmbH, PEO603). The SiNWs were subsequently grown at a temperature of 420 °C with a H_2_:SiH_4_ gas flow mixture of 10:1 and at a pressure of 150 mbar for 40 min.

### Positive electrode preparation

For the positive electrode, carbon as a porous conducting sulfur host helps to overcome the electrically insulating nature of the active material species (S_8_, Li_2_S_2_/Li_2_S)^50^. The sulfur loading was increased up to 65 wt.% in a S/C composite stack with long cycling duration^51^. The cathodes were prepared by a slightly adapted procedure from Bauer *et al*.^52^ mixing sulfur (Sigma Aldrich, 99.5%), conductive carbon black (Orion carbon) and mild grinding in a mortar mill (Fritsch Pulverisette 2) for 10 min. Subsequently, a water-based binder solution of carboxymethyl cellulose (CMC, MTI Corporation) and styrene-butadiene rubber (SBR, Targray) (ratio CMC:SBR = 1:1, m/m) was added and the slurry was again treated for 15 min in the mill. The weight ratio of sulfur:carbon:CMC:SBR in the resulting slurry was 65:30:2.5:2.5. The slurry was casted onto aluminum foil (MTI Corporation, >99.9%) via the doctor-blade coating technique. Drying of the as-prepared positive electrode sheets consisted of two steps, heating with 2 °C/min to 80 °C and thermal treatment at 80 °C for 1.5 h.

### Cell Assembly and electrochemical testing

**Figure 12:**
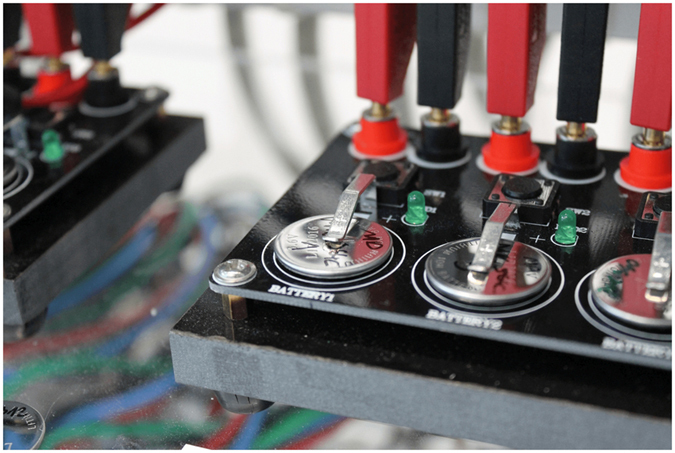
Battery cell test setup for electrochemical characterization of coin cells.

Both half and full-cells with SiNW/C composite electrodes (diameter 12 mm) have been prepared and characterized. Half-cell setups are assembled as coin cells (MTI Corp., CR2016) in an argon-filled glove box (MBraun, <0.1 ppm O_2_ and H_2_O). Metallic lithium (Pi-Kem, 99.0%, diameter 15.6 mm, thickness 250 μm) was used as negative electrode. Due to the respective potential vs. Li/Li^+^, the SiNW/C composite acts as positive electrode. The electrodes were separated by one layer of a polypropylene sheet (Celgard 2500, diameter 19 mm). The electrolyte consisted of 1 M lithium bis-(trifluoromethyl-sulfonyl)-imide (LiTFSI, Aldrich, 99.95%), 0.25 M lithium nitrate (LiNO_3_, Alfa Aesar, 99.98%, anhydrous) in 1,2-dimethoxy ethane (DME, Sigma Aldrich, 99.5%, anhydrous) and 1,3-dioxolane (DOL, Sigma Aldrich, 99.8%, anhydrous). The ratio of the solvents was 1:1 (v/v). The cells were cycled at room temperature with a BaSyTec Cell Test System (CTS). Figure 12 shows the battery in the test system. The lower and upper cut-off voltage was set to 0.01 and 1.0 V vs. Li/Li^+^, respectively. Due to the unknown maximum capacity, a constant area current of 0.5 mA/cm^2^ was used to evaluate the available capacity. 40 μl of the electrolyte were added to all coin cells. All chemicals except for LiTFSI, DME, and DOL were used as received. To remove residual water, LiTFSI was dried at 120 °C under vacuum for 24 h before use. DME and DOL were dried and stored over a 3 Å molecular sieve. All samples exhibited a Coulomb efficiency above 98% until cell failure.

For the Li/S full-cell setup, the SiNW/C composite electrode was prelithiated within two full cycles and subsequently discharged in coin cells according to the testing conditions described above. The prelithiated SiNW/C composite electrode was disassembled and rinsed with a solvent mixture of DME/DOL 1:1 (v/v) and reassembled as negative electrode vs. the sulfur/carbon composite positive electrode (65 wt.% sulfur content, diameter 12 mm) using the same electrolyte (12 μl/mg_sulfur_) and separator taken for the previously described half-cells. Due to the potential of the lithiated silicon anode, the cut-off-voltages were changed to 1.3 and 2.6 V. Galvanostatic cycling with potential limitation was carried out at a current of 1/5 C (1 C means full charging in 1 h) at room temperature.

### Physical characterization methods

The SiNW/C composite electrode was investigated by electron microscopy, X-ray diffraction and energy dispersive X-ray spectroscopy before and after integration into a battery stack in order to identify the mechanisms causing the capacity fade during cycling. Scanning electron micrographs (SEM) where taken on a ZEISS GEMINI LEO 1560 with Bruker in-lens detector, 5 kV acceleration voltage and a typical operating distance of 1 cm. X-ray powder diffraction (XRD) experiments were performed in transmission geometry with Co Kα_1_ radiation on an STOE Stadi P diffractometer with curved Ge(111) crystal monochromator and 6°-position sensitive detector. The Rietveld analysis was performed with the program MAUD^53^ assuming an isotropic size distribution. The Popa line broadening model was applied to determine the size and strain of the crystalline phases.

TEM investigations were performed with a FEI Tecnai F30 equipped with field emission gun and operated at 300 kV. Energy dispersive X-ray (EDXS) spectroscopy was operated in scanning mode (STEM). The selective area electron diffraction (SAED) pattern was analyzed with the program ELDISCA. For the cycled sample, a diluted HNO_3_ solution has been used to remove residuals from the electrolyte as well as the solid electrolyte interface (SEI). Prior to analysis, the samples were gently grinded and dispersed in acetone. The dispersion was dropped onto a copper mesh coated with lacey carbon film for TEM analysis.

### In-operando synchrotron XRD

**Figure 13:**
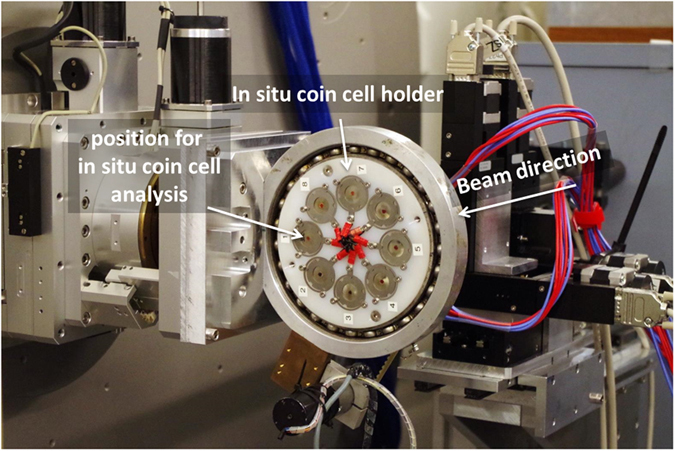
Setup for in-operando synchrotron XRD measurements at DESY Hamburg.

In-operando XRD measurements have been done at the beamline P02.1 of the 3rd generation Synchrotron Radiation Source PETRA III at DESY in Hamburg. In an 8-fold coin cell holder^54^,^55^, a modified coin cell (CR2025) with a self-made Kapton window on both electrode sides was used for the in-operando XRD measurements. Figure 13 shows the complete setup including the 8-fold coin cell holder. To avoid detection of inactive parts of the cell a hole was implemented in the stainless steel spacer. Only the alumina current collector on the cathode is detected as inactive part of the cell. The lithiated SiNWs were fabricated by discharging to 0.01 vs Li/Li^+^ at 0.5 mA and assembled vs. the S/C cathode. The beam energy was fixed to 60 keV, which ensures negligible polarization effects and very low mass absorption coefficients for most elements. A 16-inch two-dimensional flat panel detector of the XRD 1621 N ES Series (PerkinElmer, 2048 × 2048 pixels, pixel size 200 μm) was used for recording the diffraction patterns. The exposure time was set to 30 s and a previously made 30 s dark image. Detector calibration and two-dimensional image integration were realized by a LaB_6_ NIST standard and the software *Fit2D*^56^. Further details about the setup, the beam optics, the monochromators and experimental possibilities at the high-energy beamlines at P02 are reported elsewhere^57^.

## Additional Information

**How to cite this article**: Krause, A. *et al*. High Area Capacity Lithium-Sulfur Full-cell Battery with Prelitiathed Silicon Nanowire-Carbon Anodes for Long Cycling Stability. *Sci. Rep.* **6**, 27982; doi: 10.1038/srep27982 (2016).

## Supplementary Material

Supplementary Information
