# Supplementary material for: High Area Capacity Lithium-Sulfur Full-cell Battery with Prelitiathed Silicon Nanowire-Carbon Anodes for Long Cycling Stability
Source: Sci Rep. 2016 Jun 20;6:27982. doi: 10.1038/srep27982 (PMC4913245; doi:10.1038/srep27982)
Supplement: Supplementary Information [file srep27982-s1.pdf]

SUPPLEMENTARY INFORMATION

High Area Capacity Lithium-Sulfur Full-cell Battery with Prelitiathed Silicon Nanowire-Carbon Anodes for Long Cycling Stability

Andreas Krause[1,2], Susanne Dörfler[3,4], Markus Piwko[3], Florian M. Wisser[4], Tony Jaumann[5], Eike Ahrens[5,6], Lars Giebeler[5,6], Holger Althues[3], Stefan Schädlich[3], Julia Grothe[4], Andrea Jeffery[1], Matthias Grube[1], Jan Brückner[3], Jan Martin[4], Jürgen Eckert[7,8], Stefan Kaskel[3,4], Thomas Mikolajick[1,2,9], Walter M. Weber[1,2]*

[1] Namlab gGmbH, 01187 Dresden, Germany

[2] Center for Advancing Electronics Dresden (CfAED), TU Dresden, Dresden, Germany

[3] Fraunhofer Institute for Material and Beam Technology (IWS), Winterbergstr. 28, 01277 Dresden, Germany

[4] Department for Inorganic Chemistry I, TU Dresden, Dresden, Germany

[5] Leibniz Institute for Solid State and Materials Research (IFW) Dresden e.V., Helmholtzstr. 20, 01069 Dresden, Germany

[6] Institut für Werkstoffwissenschaft, TU Dresden, Helmholtzstr. 7, 01069 Dresden, Germany

[7] Erich Schmid Institute of Materials Science, Austrian Academy of Sciences, A-8700 Leoben, Austria

[8] Department Materials Physics, Montanuniversität Leoben, Jahnstraße 12, A-8700 Leoben, Austria

[9] Institute of Semiconductor and Microsystems Technology, TU Dresden, 01062 Dresden, Germany

* Andreas.Krause@namlab.com

Au precursor development

Au catalyst particles are the most convenient materials for the catalysis of SiNW growth on various substrates[1]. The elements Si and Au generate a eutectic at approximately 360 °C [2] being lower for nanoscaled particles. In order to realize high areal capacities of the anode, as required in practical applications, a high density of SiNWs is needed. For that, high Au nanoparticle densities with low diameter are required on the surface of the substrate. A three-dimensional carbon mesh has been selected as light-weight carbon-based substrate and current collector (Freudenberg FCCT SE & Co. KG, Y0200)[3] increasing the usable surface by tenfold. Nevertheless physical deposition techniques do not allow a homogeneous coverage due to shadowing effects in the 3D mesh. Moreover, commercially available synthesized Au nanoparticles are expensive and the removal of surfactants prior to the nanowire growth is critical, as remnants / contamination can be electrochemically active or can hinder nanowire nucleation. Therefore, developing a stable and recyclable Au precursor enables a resource efficient gold catalyst seed application.

The developed precursor consists of 5.822 g (0.03 mol) citric acid (Acros Organics, anhydrous, 99 %) and is dissolved at 90°C in 3.804 g (0.06 mol) ethylene glycol (Sigma Aldrich, 99.5 %). Subsequently 6 ml of half concentrated HCl (Sigma Aldrich, p.a.) were added and the solution was stirred for 1 h at 90°C and cooled to room temperature. In a typical procedure 0.475 g of the polymeric solution were diluted into 6.5 ml of ethanol (VWR, absolute) and 2.3 ml of a 0.2 M aqueous $HAuCl_4$ (ChemPur, 50 % Au) were added.

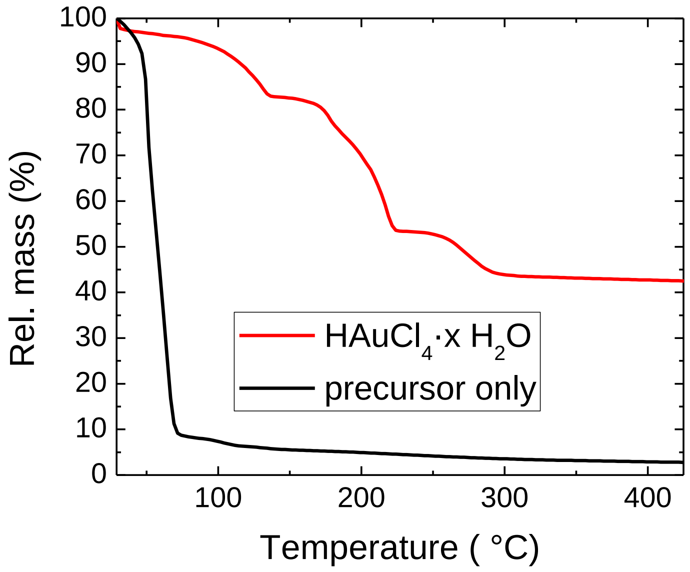

Figure S1. Thermogravimetric measurement of the gold precursor solution. At 300 °C, all residuals of the precursor are removed and gold nanoparticles are formed on the surface.

The decomposition of the precursor solution (**Figure S1**) occurs below 250 °C. In contrast pure auric acid shows an additional decomposition step around 250 to 300 °C which corresponds to the decomposition of Au(I)Cl into elemental Au. Thus due to the reducing effect of the carboxylic groups in the polymeric precursor, the reduction of Au(III) occurs at much lower temperatures.

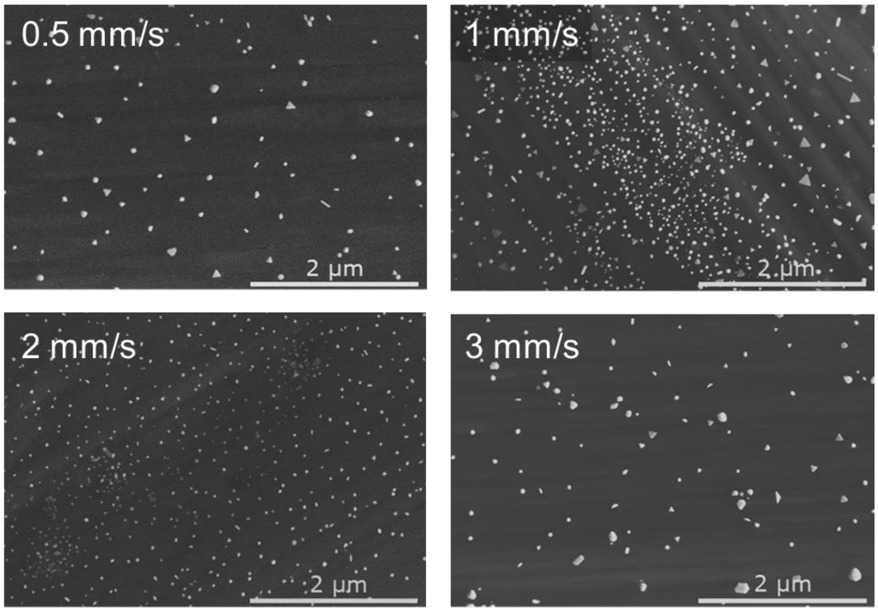

Figure S2. SEM images of carbon fiber surface after the deposition of Au with different extraction speed and the formation of Au nanoparticles. Above 2 mm/s, larger particles with sizes of more than 100 nm diameter are formed.

Prior to dip-coating, the carbon meshes were activated using an argon/oxygen plasma (Kinpen plasma pen, Neoplas Tools) in order to make the surface more hydrophilic. For a typical dip-coating procedure of the carbon mesh by a gold catalyst layer, the substrates were immersed in the precursor solution for 30 s and removed by applying a constant lifting speed of 1 or 2 mm/s. Subsequently, they were slowly annealed (2 K/min) to 200 °C and thermally treated for 2 h at 200 °C. Different Au nanoparticle sizes and densities in dependence of lifting speed are shown in **Figure S2**. Thicker precursor layers from faster precursor extraction speeds (above 2 mm/s) result in much larger Au clusters. Typically they do not contribute to the Si nanowire growth.

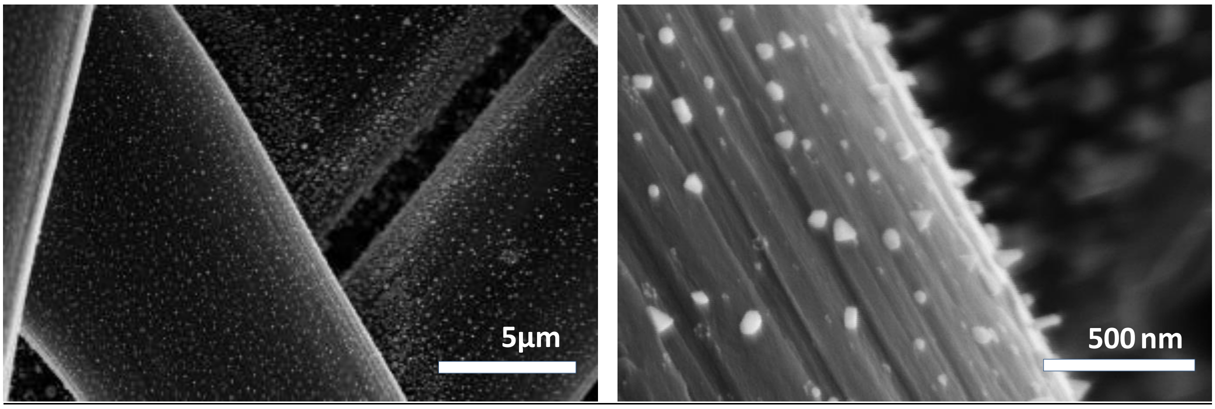

Figure S3. Au nanoparticles on the three-dimensional carbon fiber. The particles are homogenously distributed on top of the carbon surface with a diameter of approximately 50 nm. The lifting speed has been set to 2 mm/s.

Typical particle sizes on the carbon meshes are in a range from 30-80 nm for the 2 mm/s lifting speed (**Figure S3**). Remains of ultrathin Au layers on top of the carbon surface are not visible, but cannot be excluded in the SEM images. The hydrophobic nature of the carbon surface seems to be beneficial for the formation of the Au layer. In contrast the growth on flat Si pieces as testing substrate (with native oxide coverage) shows complete inhomogeneous Au layers due to the different wetting behavior of the polymeric precursor.

Physical characterization of as-grown SiNWs on carbon mesh

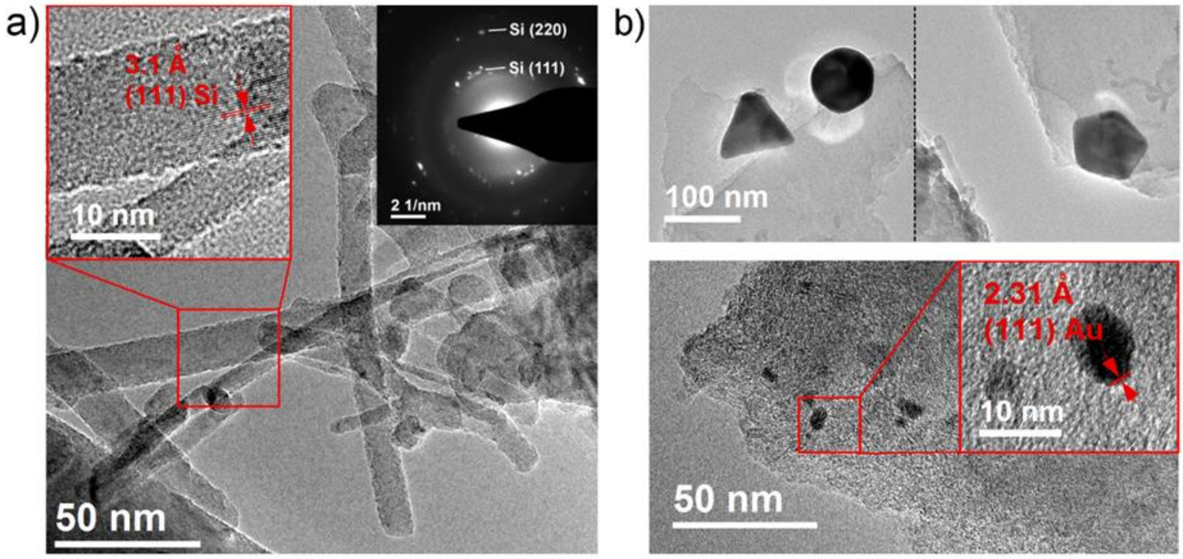

Figure S4: TEM bright-field images and SAED pattern of SiNW deposited on the carbon support prior to lithiation a) with focus on the SiNW and b) with focus on the gold nanoparticles (EDX analysis of area b is available in the support information).

TEM bright field images (**Figure S4**a) show various SiNWs with diameters between 5 – 30 nm of different length which are not the length of the as prepared wires due to cracking during sample preparation. Si nanoparticles with diameters of about 10 – 40 nm are observed as agglomerates on the carbon support. High-resolution images (Figure S4a) reveal a crystalline Si structure with space group $Fd\bar{3}m$ indicated by lattice planes with a d-spacing of 3.1 Å for the Si {111} plane. The crystalline structure is verified by selected area electron diffraction (SAED) pattern. The indexing of the typical ring pattern fits well with the lattice parameters of cubic Si. In addition, the rings of amorphous carbon from the supporting mesh are present. Several Au nanoparticles with diameters of 5 – 80 nm are observed in Figure S4b represented by noticeable dark areas in the bright field image. The shape varies from spherical to triangular and is consistent with results from scanning electron microscopy. The high-resolution images evidence the crystalline Au particles due to a determined d-spacing of 2.31 Å assigned to the {111} Au plane (EDXS analysis see **Figure S5**).

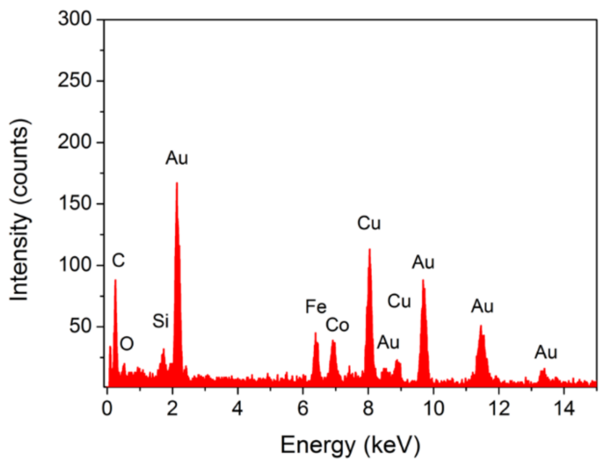

Figure S5. EDX analysis of the gold nanoparticles observed in the TEM bright-field image. The Cu, Fe and Co signals originate from the Cu-grid. Only low amounts of Si are present in the vicinity of the larger Au nanoparticles.

<u>In-operando XRD measurements</u>

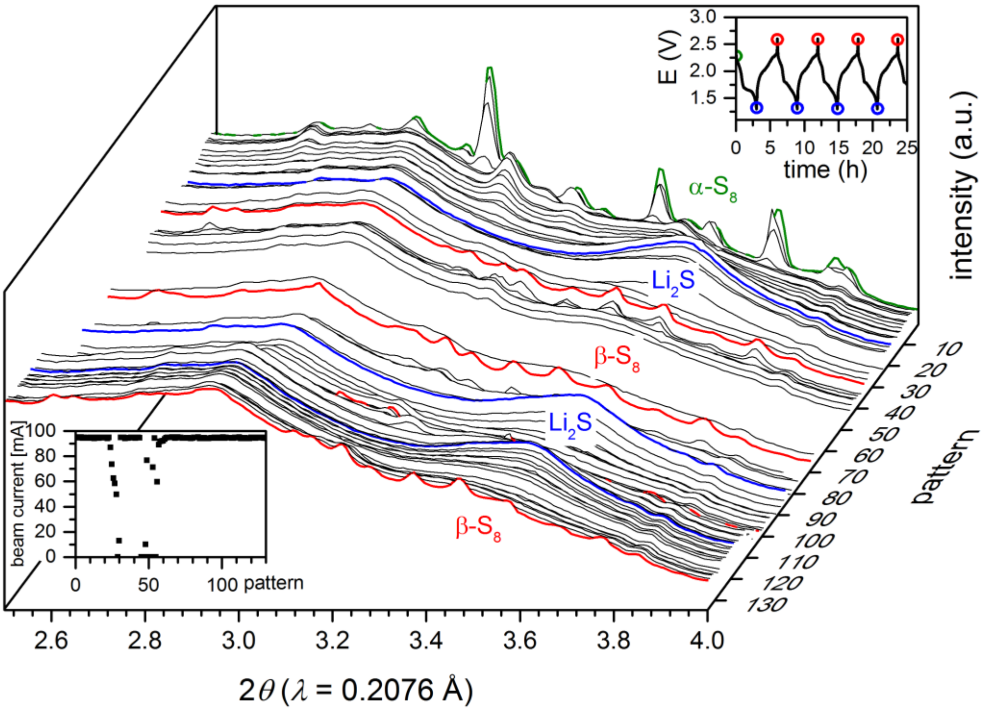

Figure S6. In-operando synchrotron powder diffraction data of the cycled lithiated SiNW – S full-cell at 1/5 C. Corresponding electrochemistry with assigned position of the colored XRD pattern (inset top right) and beam current showing the beam bumps between pattern 20 and 65 (inset bottom left).

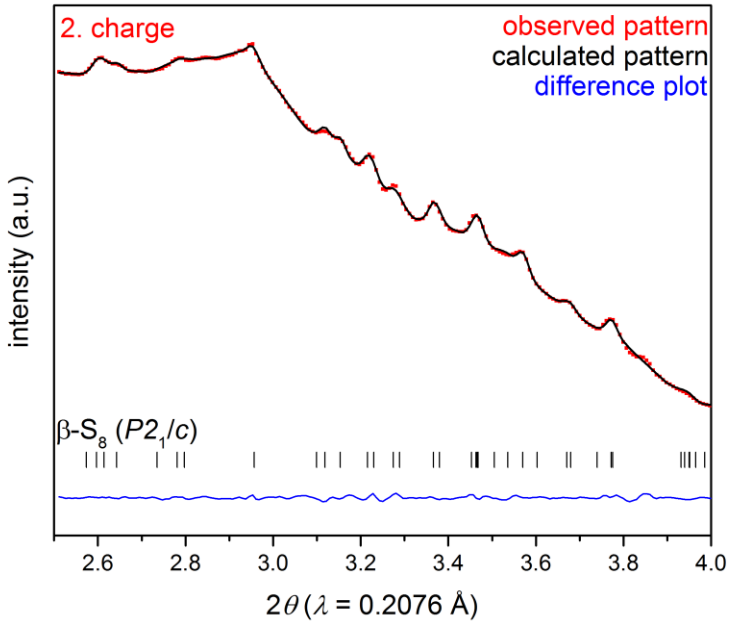

Figure S7. In-operando synchrotron powder diffraction data of the cycled lithiated SiNW – S full-cell at 1/5 C. Rietveld analysis of the pattern taken at the end of the 2.charge. The β-sulfur structure is refined.

References

1. Wagner, R. S. & Ellis, W. C. VAPOR-LIQUID-SOLID MECHANISM OF SINGLE CRYSTAL GROWTH. *Appl. Phys. Lett.* **4,** 89–90 (1964).
2. Okamoto, H. & Massalski, T. B. The Au−Si (Gold-Silicon) system. *Bull. Alloy Phase Diagr.* **4,** 190–198 (1983).
3. Brückner, J. *et al.* Carbon-Based Anodes for Lithium Sulfur Full Cells with High Cycle Stability. *Adv. Funct. Mater.* **24,** 1284–1289 (2014).
